# Effect of Polymer Infiltration on the Flexural Behavior of β-Tricalcium Phosphate Robocast Scaffolds

**DOI:** 10.3390/ma7054001

**Published:** 2014-05-21

**Authors:** Francisco J. Martínez-Vázquez, Antonia Pajares, Fernando Guiberteau, Pedro Miranda

**Affiliations:** Department of Mechanical, Energy and Materials Engineering, University of Extremadura, Avda de Elvas s/n., 06006 Badajoz, Spain; E-Mails: chico@materiales.unex.es (F.J.M.-V.); apajares@unex.es (A.P.); guiberto@unex.es (F.G.)

**Keywords:** polymer impregnation, robocasting, scaffolds, β-tricalcium phosphate, bending strength, toughness

## Abstract

The influence of polymer infiltration on the flexural strength and toughness of β-tricalcium phosphate (β-TCP) scaffolds fabricated by robocasting (direct-write assembly) is analyzed. Porous structures consisting of a tetragonal three-dimensional lattice of interpenetrating rods were impregnated with biodegradable polymers (poly(lactic acid) (PLA) and poly(ε-caprolactone) (PCL)) by immersion of the structure in a polymer melt. Infiltration increased the flexural strength of these model scaffolds by a factor of 5 (PCL) or 22 (PLA), an enhancement considerably greater than that reported for compression strength of analogue materials. The greater strength improvement in bending was attributed to a more effective transfer of stress to the polymer under this solicitation since the degree of strengthening associated to the sealing of precursor flaws in the ceramic rod surfaces should remain unaltered. Impregnation with either polymer also improved further than in compression the fracture energy of the scaffolds (by more than two orders of magnitude). This increase is associated to the extraordinary strengthening provided by impregnation and to a crack bridging toughening mechanism produced by polymer fibrils.

## 1. Introduction

Biodegradable scaffolds are called to be the long-term future materials for bone tissue engineering applications. These materials provide structural support and stimulate bone regeneration, being used as temporary structures providing mechanical stability and a suitable (osteoconductive/osteoinductive) extracellular matrix to promote the generation of newly-formed living tissue, eventually leaving a fully healed lesion.

Porous bioceramic scaffolds are widely regarded as promising candidates for bone tissue regeneration [1]. Hydroxyapatite (HA) and tricalcium phosphates (TCPs) are the most commonly used materials in clinical applications because of their biocompatibility, osteoconductivity, bioactivity, bioresorbability, and their chemical similarity to the mineral phase of bone [2–4].

The pore architecture in such materials must exhibit an appropriate level of interconnectivity [5–7] to allow vascularization and diffusion of nutrients in order to promote cell ingrowth. This requirement implies a large total porosity for those scaffolds that are fabricated by conventional fabrication techniques [8–10]. Consequently, such bioceramic structures are extremely brittle, which prevents a more widespread usage of these materials. This hurdle is partially overcome by additive manufacturing (also known as rapid prototyping or solid freeform fabrication) techniques as they allow a precise control over scaffold external—which enable the fabrication of patient-specific scaffolds from medical scans—and internal geometry, making it possible to optimize the pore architecture, by maximizing pore interconnectivity while keeping the total porosity to a minimum. For instance, robocasting fabrication has been shown to provide a significant improvement in the compressive strength of β-tricalcium phosphate (β-TCP) scaffolds [11,12]. Nonetheless, the strength of these TCP robocast scaffolds, not to mention their toughness, remains far short of bone properties with the same level of porosity.

The impregnation of porous structures with biodegradable polymers (polycaprolactone and polylactic acid) has been proved to be an effective means to further improve their compressive strength, and also their toughness, to levels close to those of cortical bone with the same density [13–18]. As demonstrated in previous works, the two mechanisms involved on the strengthening of this type of structures are defect healing and stress shielding [16–18]. Briefly, in the former, the polymer acts as an sealing agent bonding together the walls of pre-existing flaws in the ceramic scaffold surfaces and precluding the initiation of cracks, and in the latter it helps sustaining the applied load, provided that it has a sufficiently high elastic modulus, as occurs notably in the case of poly(lactic acid). On other hand, the toughening effect reported previously was attributed to the strengthening produced by polymer infiltration and to an increase in ductility due to the incorporation of a continuous polymeric phase [14,16–18].

However, implants are not solely subjected to compressive forces, but to complex mechanical solicitations (not only after implantation but also during surgery). In spite of that, most of existing studies have focused on the evaluation of the compressive behavior of scaffolds [19–24], without much information regarding their flexural performance. Indeed, there are only a few studies on this topic, dealing mostly with nearly dense ceramic samples [25–28] or randomly porous structures [14,15,29,30], but none in the case of the promising polymer/ceramic robocast structures. There are also no previous studies analyzing the fracture modes developed on robocast structures under bending. Thus, the present work seeks to redress these deficiencies and to analyze, both at micro- and macroscales, the effect on flexural behavior of completely filling the macroscopic porosity of a β-TCP robocast scaffold with two different biodegradable polymers, poly(lactic acid) (PLA) and poly(e-caprolactone) (PCL), by melt impregnation. For this purpose, strength and strain energy density of bare and hybrid scaffolds measured in four-point bending tests are compared. In addition, numerical simulations (FEM) of the four-point bending tests are used to justify, as done previously in compression [12,16,31], the *in situ* observed fracture modes, while SEM observations of fracture surfaces are used as the basis for the discussion of polymer toughening effects. Eventually, the results are compared with the bending strength and toughness of bone.

## 2. Results and Discussion

### 2.1. Results

**Figure 1.**
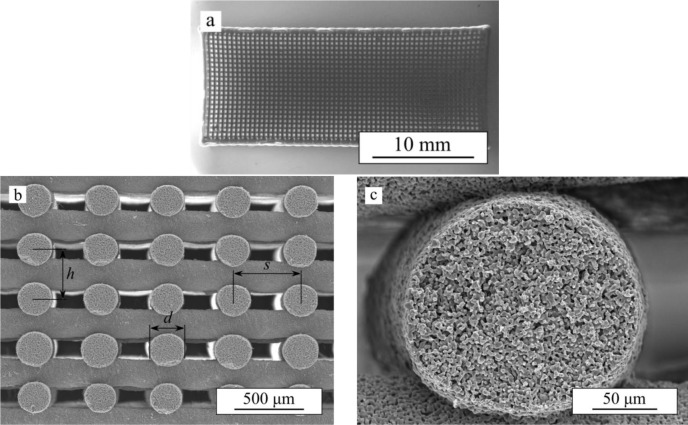
As-received β-TCP scaffolds: (**a**) printing-plane optical image; (**b**,**c**) SEM micrographs of transversal sections.

Figure 1 shows an optical image of the as-received β-TCP robocast scaffolds and SEM micrographs of representative cut specimens. Both the macroscopic, pre-designed porosity (Figure 1a,b) and the internal rod porosity (Figure 1c) of the bare scaffolds are apparent. For the sake of simplicity, we will from now on refer to the former as macroporosity and to the latter as microporosity. The average dimensions of the bioceramic structure as measured from SEM images are: rod diameter, *d* = 215 ± 8 μm, center-to-center rod spacing, *s* = 436 ± 9 μm, and center-to-center layer spacing, *h* = 162 ± 5 μm. Density measurements determined a total porosity of ~68% in the bare β-TCP scaffolds of which ~64% was open porosity, and, thus, accessible to the polymer.

**Figure 2.**
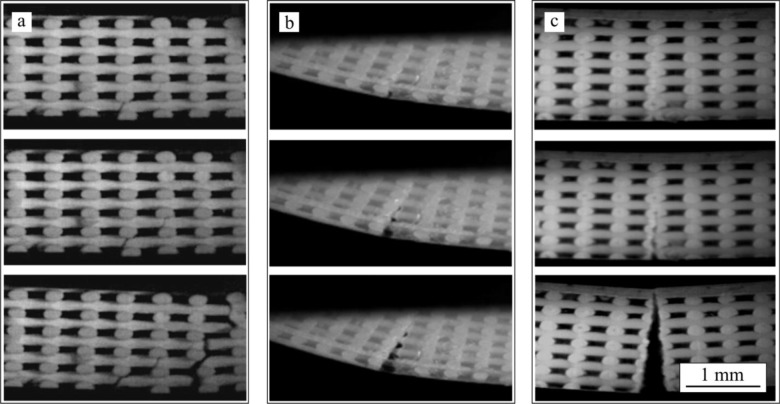
Optical image sequences captured *in situ* showing the evolution of damage during 4-point bending tests performed in the direction orthogonal to the rods (rods appear aligned from left to right and perpendicular to the image) on (**a**) bare β-TCP; (**b**) PCL-impregnated; and (**c**) PLA-impregnated scaffolds.

**Figure 3.**
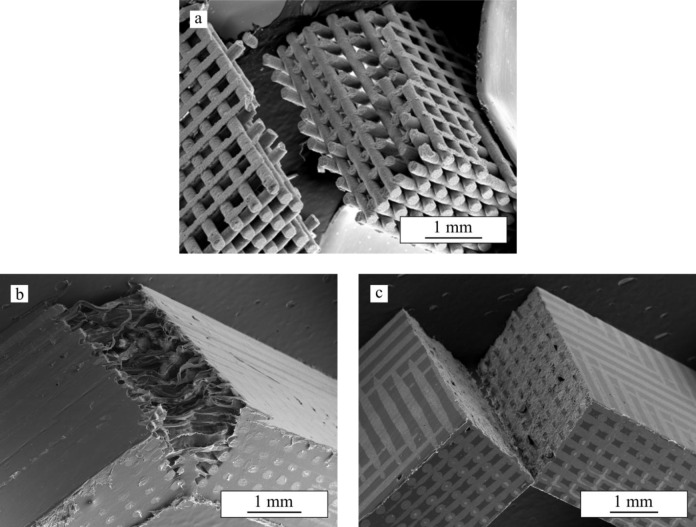
SEM micrograph showing the fracture surface on (**a**) bare TCP; (**b**) TCP/PCL; and (**c**) TCP/PLA samples after a 4-point bending test.

Figure 2 shows series of *in situ* images taken during bending tests performed on bare β-TCP scaffolds and on the same structures after impregnation with PCL (TCP/PCL) and PLA (TCP/PLA). Ceramic rods are aligned perpendicular to the image, from now on referred as transversal rods or from left to right (longitudinal rods). For bare β-TCP, (Figure 2a), at a certain load a crack pops in at the bottom of the porous structure and propagates quickly, within a few milliseconds, through the height of the specimen breaking it in two parts. As is apparent in these images, the crack cuts just the longitudinal rods, close to the contact with the transversal rods. However, the crack path is not straight but rugged. All this can be more clearly seen in the SEM micrograph of Figure 3a, which shows the fracture surface of the bare scaffold after the 4-point bending test.

In the TCP/PCL structure (see sequence in Figure 2b), the crack similarly cuts just the longitudinal ceramic rods but propagates much straighter and slower (>1 s) than in bare β-TCP specimens. The polymer is now able to hold together the structure long after the ceramic skeleton fails, and large stretched polymer filaments are evident bridging the crack walls together even after very large strains. This can be clearly observed in the SEM micrograph of the structure after bending test (Figure 3b). Indeed, unlike what happens in bare β-TCP, TCP/PCL specimens are not broken in two during the test.

On the contrary, in the case of TCP/PLA (Figure 2c), the fracture is more brittle and the crack propagates quickly although not as fast as in the bare scaffolds (~10 ms *vs.* ~1 ms). The morphology of the crack is also different in that it now cuts not only the longitudinal ceramic rods transversally, but also the transversal ones along their axis. The crack path is straight as in the TCP/PCL specimens, but there is no evidence of polymer filaments bridging the crack. All these features are confirmed in the SEM micrograph of Figure 3c.

**Figure 4.**
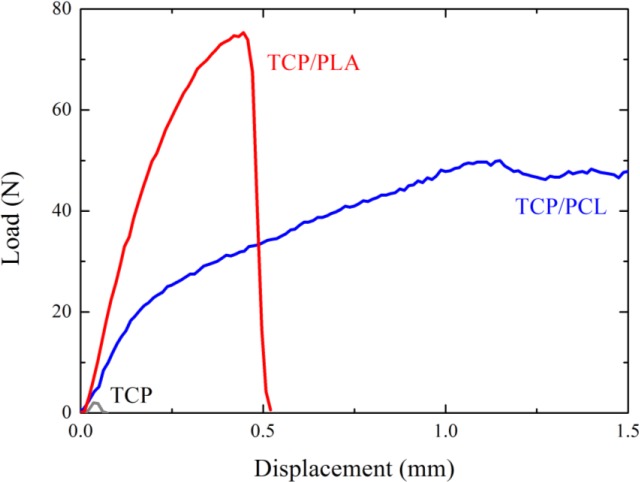
Representative load-displacement curves obtained from 4-point bending tests performed on bare TCP, and on TCP/PCL and TCP/PLA hybrid samples.

Figure 4 shows characteristic load-displacement curves from flexural tests performed on β-TCP bare and impregnated scaffolds. In the curves corresponding to β-TCP and TCP/PLA, the significant drop in the load is associated with crack pop-in. The onset of this crack corresponds to the maximum load, and therefore determines the bending strength of the scaffold (see Experimental section). Conversely, curves for PCL-impregnated structures do not exhibit a maximum, but just a clear slope decrease, which is attributed to the fracture of the ceramic skeleton. The transition load, calculated from the intersection of the linear best fits to data before and after the transition, was used to determine the flexural strength of the composite structure. A significant improvement in the bending strength and toughness of the TCP scaffolds is apparent upon infiltration of either PLA or PCL.

**Figure 5.**
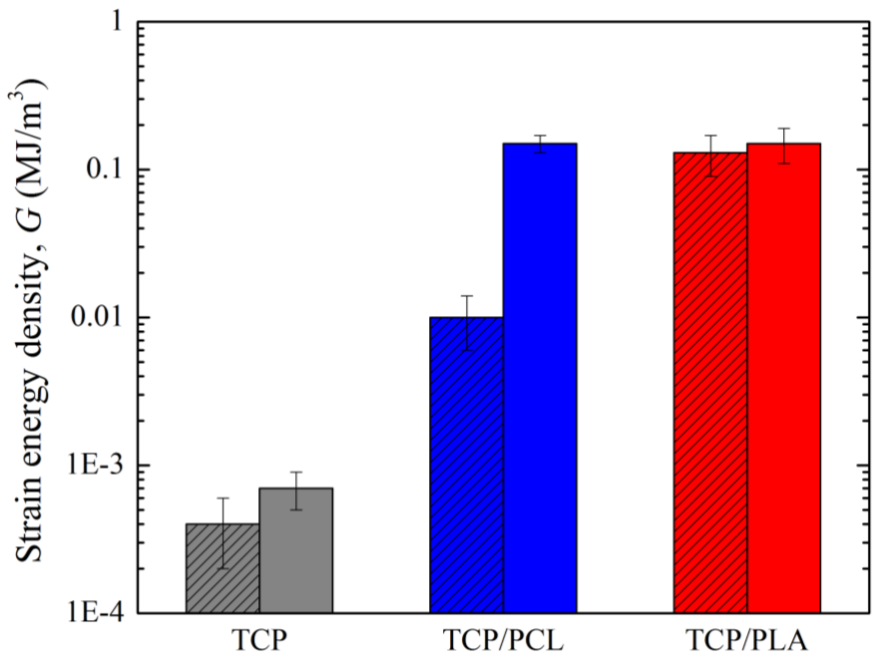
Strain energy density, calculated from bending test for scaffolds before and after impregnation with PCL and PLA. The patterned bars represent *G*_max_ values, while the solid ones correspond to *G*_1_. Average values are shown with standard deviations as errors.

Data for fracture energy density are included in Figure 5. For bare-TCP, hardly any difference is apparent between the fracture energy density at maximum strength (*G*_max_) and at 1 mm stroke (*G*_1_), denoting a lack of any actual significant ductility in the bare scaffold as has already been indicated. The impregnation of scaffolds enhanced *G*_max_ by at least one order of magnitude, and *G*_1_—once the ceramic skeleton has failed—by more than two orders of magnitude. Although in this latter case identical absolute values were fortuitously obtained for both composites structures, it is worth mentioning that TCP/PCL specimens are in fact significantly tougher, since no evidence of load drop was detected even at greater displacements. This is due to the intrinsic greater ductility of PCL, which is able to provide some linkage between the crack walls, as shown in Figures 2b and 3b, while TCP/PLA samples break in a somewhat brittle manner (Figure 3c) with virtually equal *G*_1_ and *G*_max_ values.

**Figure 6.**
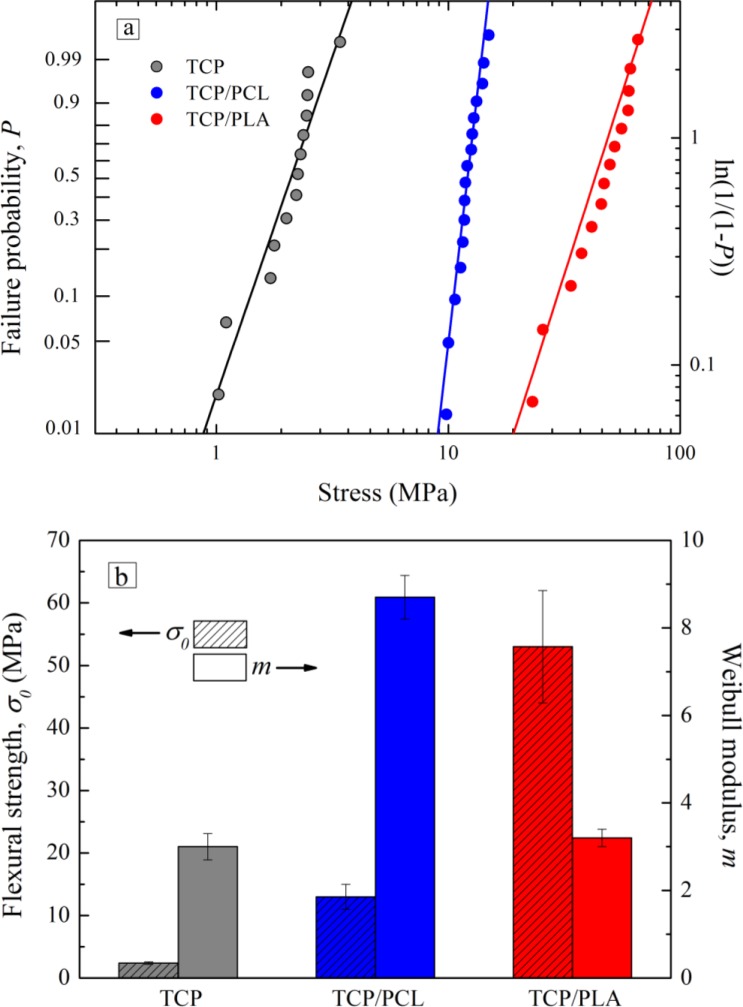
(**a**) Weibull bending strength plots for TCP, TCP/PCL, and TCP/PLA structure; (**b**) Central values, *σ*_0_, and Weibull moduli; *m*, with standard errors, for each structure.

The strengthening effect of polymer infiltration can be observed more clearly in the Weibull plots of Figure 6a. This plot shows the failure probability as a function of applied bending stress. The straight lines are the best fits to experimental data using the Weibull probability function [32]:

$$P=1-e^{-{\left(\sigma /\sigma_{0}\right)}^{m}}$$ (1)

with *P* being the failure probability; and where the Weibull modulus, *m*; and central value, *σ*_0_, are adjustable parameters whose values are summarized in Figure 6b. The low bending strength and reliability of bare scaffolds confirm the brittleness of these porous TCP structures.

The strengthening factors calculated from these data are 5 ± 1 after impregnation with PCL and 22 ± 6 in the case of PLA infiltrations. These factors are higher than the corresponding values reported for compression of analogue hybrid structures [16]. On the other hand, reliability also increases upon impregnation with PCL with respect to bare structures, as evidenced by their Weibull modulus (*m* = 8.7 ± 0.5 *vs.* 3.0 ± 0.3), but does not change after PLA infiltration (*m* = 3.2 ± 0.2).

**Figure 7.**
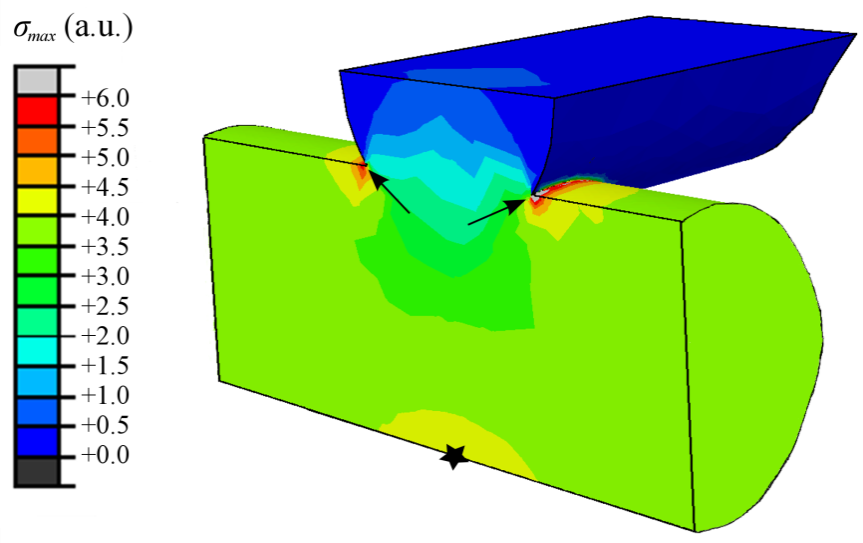
FEM-generated stress contours corresponding to the maximum tensile stress *σ*_max_ at the external surfaces of TCP lowermost rods during 4-point bending test on a bare TCP scaffold.

The FEM calculated stress contours corresponding to the maximum tensile stress, *σ*_max_, at the surface of the two lowermost rod layers of the bare scaffold are shown in Figure 7. The maximum tensile stresses are found at the surfaces of the lowermost longitudinal rods in the sample. There are two relative maxima, one (indicated by arrows in Figure 7) located at the upper surface of the rods close to the joints with the transversal rods and other at the lower surface, right below the contact with the transversal rods (designated by a star symbol in Figure 7). The location of the maxima does not change on infiltrated structures although the magnitude of the stresses does—the results are not shown to avoid misleading comparisons between systems, as the FEM data is not considered quantitatively reliable due to computational limitations.

### 2.2. Discussion

Since this work constitutes the first study in analysing the damage generated on robocast structures under bending stresses, it is worth starting the discussion by justifying the observed fracture modes. On bare scaffolds (Figure 2a) and PCL-impregnated structures (Figure 2b), the experimental observations of crack initiation at, and preferred propagation through, the longitudinal rods close to the joints with the transversal rods are in perfect concordance with the location of the maximum tensile stresses (indicated by arrows in the FEM contours of Figure 7). In the TCP/PCL structure, the need to break the polymer infiltrate as the crack propagates prevents it from taking the irregular path observed in the bare structures in favour of the straighter vertical route through the joint-adjacent maxima.

On the contrary, the crack pattern observed in TCP/PLA structures through a plane bisecting both the longitudinal and the transversal rods (Figure 2c) cannot be explained solely in the light of the stress field. It is true that there is a local relative maxima of *σ*_max_ located at the lower surface of the longitudinal rods, below the contact with the transversal rods, (designated by a star in Figure 7), which could facilitate crack initiation at the aforementioned planes. However, this does not explain why the crack ignores all the stress maxima located close to the rods’ intersections.

**Figure 8.**
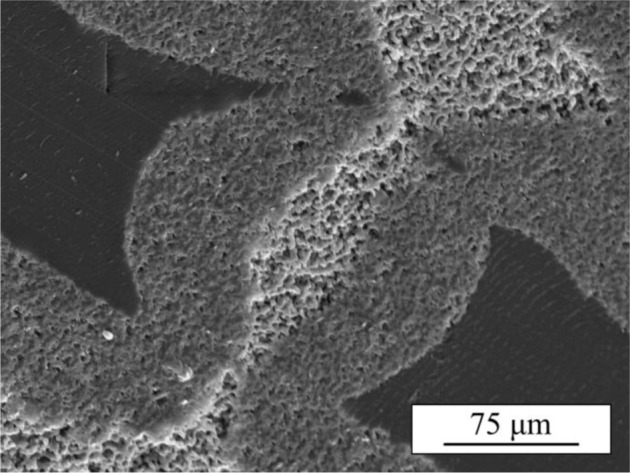
SEM micrograph showing the incomplete impregnation of rods on a TCP/PLA bending test sample.

A close inspection of TCP/PLA bending samples reveals that infiltration of the in-rod porosity by PLA in these samples was incomplete, as a consequence of the high viscosity of PLA, leaving some empty pores at the cores of many a rod, especially at the interior of the samples (Figure 8). These unhealed defects lower the intrinsic strength of the rods in those regions, facilitating the crack propagation through the rods’ cores and effectively reducing the overall strength of the composite structure.

This incomplete healing of rods’ micro-defects is also responsible for the notable reduction in the reliability of TCP/PLA structures compared to that of TCP/PCL that can be appreciated in Figure 6b. Indeed, the Weibull modulus of TCP/PLA structures is similar to that of bare scaffolds, demonstrating that they share a similar flaw distribution (at least in terms of width). This suggests that defect healing, one of the two strengthening mechanisms under compressive stresses, might be virtually inactive in these TCP/PLA structures.

Nonetheless, the results in Figure 6b reveal that the improvement on the strength of robocast β-TCP scaffolds upon impregnation is even higher under bending stresses than it is in compression. Indeed, the strengthening factors calculated here, 5 ± 1 after impregnation with PCL and 22 ± 6 in the case of PLA infiltrations, are twice the values previously reported for compression [17]. This can only be attributed to an increase in the effectiveness of the stress shielding mechanism. Certainly, defect healing effect modifies just the intrinsic strength of the rods and, thus, should not depend on the testing configuration but just on the initial flaw population, the polymer properties and the quality of the polymer infiltration. As the scaffold fabrication and melt impregnation processes used here were the same as in a previous work [17], flaw population and polymer properties should be virtually unaltered and while the quality of the infiltration can be affected by the use of larger samples, any change would be for the worse, as it is the case here for TCP/PLA scaffolds (Figure 8). Therefore, these results suggest that the stress shielding strengthening is much more significant under bending stresses than it is in compression. Since defect-healing strengthening factor in compression for PCL-impregnated samples was estimated in previous work to be 2.2 ± 0.4, a simple calculation allow one to estimate the stress-shielding strengthening factor in bending for TCP/PCL samples to be around 2, that is a 100% improvement in strength *vs.* the 20% (strengthening factor 1.2) improvement achieved in compression [18]. And in the case of PLA, as already mentioned, stress shielding is deemed to be virtually the sole responsible for the 22 ± 6 experimental strengthening factor, a value an order of magnitude higher than the stress-shielding strengthening factors (~3) estimated in compression [16].

The toughening effect of polymer impregnation is also more spectacular in bending tests than in compression [17]. According to the values reported in Figure 5, the fracture energy density increases by more than two orders of magnitude over bare structures for both polymers (*vs*. the one order of magnitude improvement achieved in compression [17]). Despite its much smaller associated strengthening, PCL-impregnated structures consume as much energy at 1 mm stroke (*G*_1_) than TCP/PLA structures. Strain energy density continues increasing much further in TCP/PCL structures since they preserve mechanical integrity even after the tests are stopped at 2 mm stroke, whereas TCP/PLA samples are broken in half at around 0.5 mm stroke (Figure 4).

**Figure 9.**
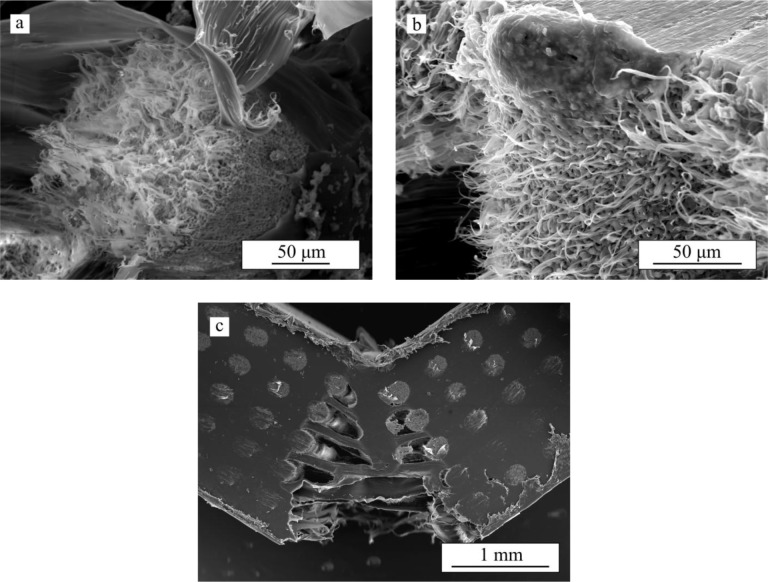
SEM micrographs of a TCP/PCL specimen after bending test: (**a**) fracture surface of longitudinal rods; (**b**) lateral surfaces of transversal rods; and (**c**) lateral view of the bended specimen. Both the micro- and macrofibrils are evident.

This extraordinary toughening provided by PCL long after cracking in the ceramic skeleton occurs is provided both by macro- and micro-fibrils (see Figure 9) that bridge the crack opening [15].

PCL microfibrils are generated from the polymer within the micropores, both at the fracture surface of longitudinal rods (Figure 9a) and at the lateral surfaces of transversal rods (Figure 9b) where ceramic/polymer detachment has to occur in order to propagate the crack. These microfibrils dissipate a large amount of energy until they break, providing a continuous crack microbridging toughening right behind the crack front. On the other hand, the macrofibrils generated from the polymer occupying the longitudinal macropore channels (Figure 9c) bridge the crack all along its wake. Thus, their contribution to energy dissipation only increases as the crack propagates. And it is precisely these macrofibrils that hold the sample together even after very large strains.

**Figure 10.**
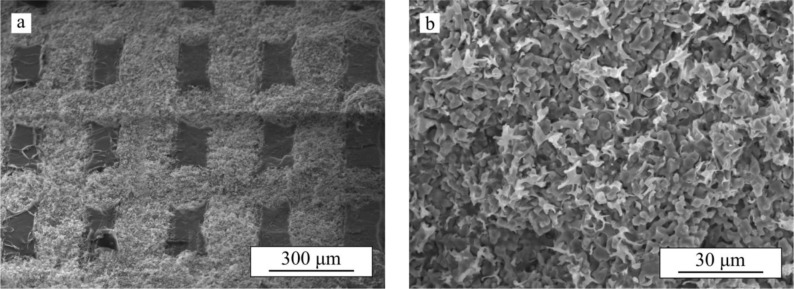
SEM micrographs of a TCP/PLA specimen after bending test: (**a**) fracture surface at low magnification; and (**b**) detail of fracture surface within a rod. Short microfibrils but no macrofibrils are evident.

On the contrary, on PLA-impregnated structures only short microfibrils from the polymer within the rods microporosity are apparent, with the polymer in the macropores breaking smoothly without significant elongation (Figure 10), evidencing the intrinsic brittleness of this polymer. Therefore, apart from a small contribution from a crack microbridging mechanism by microfibrils, the increase in fracture energy is basically through the extraordinary strengthening provided by PLA infiltration.

**Figure 11.**
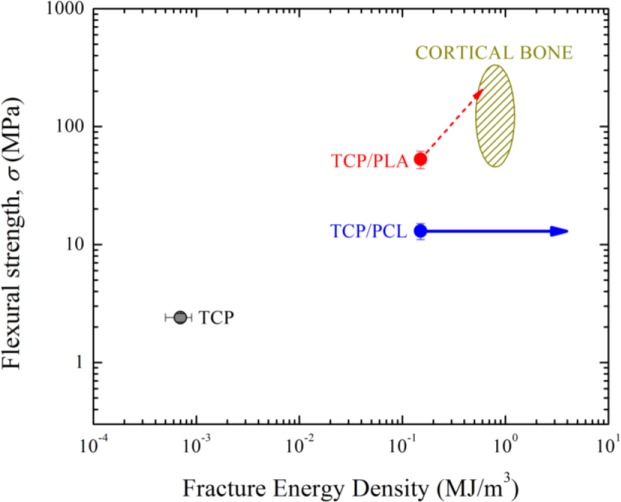
Ashby diagram flexural strength *vs.* fracture energy density comparing the properties of bare and impregnated robocast scaffold with cortical bone properties [33].

All these results highlight the critical role an infiltrating polymer can have on improving the mechanical performance of the scaffolds especially against this most deleterious type of solicitations. As can be appreciated in the Ashby diagram of Figure 11, the combination of properties achieved in these hybrid structures under bending forces is very close to that of cortical bone [33], much closer than what can be achieved with bare scaffolds. In particular, TCP/PCL composites are deemed to surpass cortical bone performance in terms of toughness (fracture energy density) since they do not actually break during the tests (indicated by a horizontal arrow in Figure 11), although unfortunately they fall somewhat short in terms of strength. On the other hand, TCP/PLA composites can closely match the performance of cortical bone in terms of the mechanical strength and, possibly, toughness—Note that the strength of TCP/PLA composites, and thereby their fracture energy density, might be increased from the values reported here by as much as 3–4 times, which is the defect-healing strengthening factors estimated in previous works for PLA [16], (this is indicated by a discontinuous arrow in Figure 11) if a full impregnation of the rod microporosity was achieved.

This bone-like performance may not be so surprising since the developed scaffolds are organic-inorganic composites just like bone, and with relatively similar calcium phosphate content (60–90 wt% for bone). What is quite surprising is that such fine performance has been achieved by using a randomly chosen geometry for the initial robocast structures and off-the-shelf materials. This suggests that even better mechanical performances could be achieved in these hybrid structures through the optimization of the pore architecture of the initial robocast scaffold and a careful selection of the individual materials forming the composite.

As a final remark, it is worth mentioning that although fully impregnated composite scaffolds lack the open macroporosity necessary to induce bone ingrowth after implantation, they remain bioactive and bioresorbable while exhibiting the best mechanical performance among this select group of materials. These dense materials might find applications on temporary implants and fixation devices, as an alternative to polymer matrix composites reinforced with ceramic particulates or fibres. While the porous robocast bioceramic structures might not be the best reinforcing phase for the polymeric matrix they would provide a greater control on biodegradation behavior and a significantly improved stiffness over a discontinuous reinforcing phase. In addition, a careful selection of the materials comprising these co-continuous ceramic/polymer structures—e.g., a polymer infiltrate with higher bioerosion rate than that of the ceramic skeleton—might induce the generation of porosity *in situ* upon implantation to enable bone in-growth and regeneration. The realization of such optimal biomaterials (bioactive, bioresorbable, with bone-like density, toughness and strength, and capable of inducing bone regeneration) remains a scientific challenge but this work significantly contributes towards this ultimate goal by providing useful information for a better understanding of the mechanical behavior of these novel type of structures.

## 3. Experimental Section

### 3.1. Starting Materials

Commercially available scaffolds (Ceramics 3D, Badajoz, Spain), with a 84 wt% β-TCP/16 wt% calcium pyrophosphate (CPP) composition were obtained, whose preparation procedure is similar to that described elsewhere [16,34]. Three-dimensional β-TCP scaffolds consisted of a tetragonal mesh of ceramic rods constructed layer by layer—each layer consisting of parallel rods, oriented orthogonally to adjacent layers—via a robotic deposition device (Aerotech A3200, 3D inks LLC, Stillwater, OK, USA). The samples were sintered at 1200 °C (heating rate 3 °C/min) for 2 h, which are the optimal sintering conditions for this particular composition [35]. The external dimensions of the scaffolds were roughly 10 × 14 × 27 mm^3^ and the internal dimensions were measured by SEM.

Density of the scaffolds was measured as total weight divided by external volume. Total porosity was determined from these measurements by considering 3.07 g/cm^3^ as theoretical density for the TCP material, and the percentage of closed porosity was measured by He-pycnometry.

### 3.2. Polymer Infiltration Process

Some composites were fabricated by melt impregnation using a procedure described in a previous work [16]. Briefly, commercial pellets of PLA (Natureworks, Minnetonka, MN, USA) or PCL (Purac, Barcelona, Spain) were melted at 227 °C and 220 °C, respectively. TCP scaffolds were then immersed in the melts, soaked for 2 h, and cooled to room temperature.

### 3.3. Mechanical Characterization

Rectangular parallelepiped specimens with dimensions of around 2 × 4 × 27 mm^3^ were cut from both bare and infiltrated structures for scanning electron microscopy (SEM) observation (S-3600N, Hitachi Ltd., Tokyo, Japan) and mechanical characterization by four-point bending tests. These experiments were carried out on a universal testing machine (AG-IS10kN, Shimadzu Corp., Kyoto, Japan), in air, at a constant crosshead speed of 6 mm/min. The load was applied in the direction perpendicular to the printing plane (*i.e*., orthogonal to the rod axes) with the contact cylinders aligned to some of the rods and perpendicular to others. The load *vs.* displacement curve was registered during the tests. Toughness was estimated as the strain energy density *G*, calculated dividing the integral of the load-displacement curve by the relevant volume of the specimen (*wtL*_2_). *G* was calculated at two stroke values: the one corresponding to the maximum load (*G*_max_) and at 1 mm (*G*_1_).

**Figure 12.**
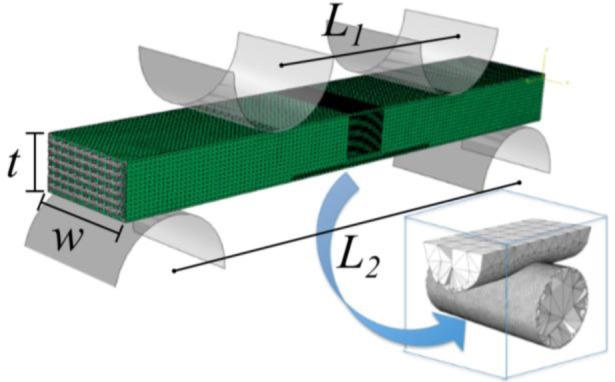
Finite element mesh used for the simulation of 4-point bending test on bare and polymer-impregnated scaffolds. The dimensions of the elements at the rod surfaces are around 100 μm, except in the central bottom region, shown in the inset, where the size is reduced to 5 μm.

The flexural strength of each structure, *σ_f_*, was calculated by the following equation:

$$\sigma_{f}=\frac{3\cdot (L_{2}-L_{1})\cdot F_{\max}}{2\cdot w\cdot t^{2}}$$ (2)

where *L*_1_ = 10 mm and *L*_2_ = 20 mm are the distances between the two upper and two lower support cylinders, respectively; *F*_max_ the maximum applied load; *w* the width of the specimen and *t* its thickness (Figure 12). A least 15 samples of each material were tested in order to get statistically reliable values.

The intrinsic mechanical properties of the materials involved in this study were evaluated using instrumented indentation (Nanotest, Micro Materials Ltd., Wrexham, UK). Berkovich indentation tests were performed on samples of the PLA and PCL polymers and on transversal sections, polished to 1 μm finish, of the rods of bare and impregnated scaffolds. For the evaluation of rod properties, single indentations were made in the center of the circular section of at least 10 rods at 1 N maximum load and 10 s dwell time. This peak load was chosen to ensure that the indented region (~40 μm wide) was large enough compared to grain size to provide meaningful information about the mechanical properties of the rods (and not of individual grains) but small enough to avoid the influence of the free surface of the rods. For the polymer measurements, multiple indentations of fixed 20 μm depth were done on polymer areas from the periphery of infiltrated scaffolds.

### 3.4. Numerical Modeling

Finite element simulations were carried out using ABAQUS/Standard^®^ software (Hibbitt, Karlsson & Sorensen, Inc., Pawtucket, RI, USA) to calculate the stress field in scaffolds under four-point bending tests. The algorithm models the flexure of a rectangular parallelepiped bare/polymer-impregnated scaffold with external dimensions 2 × 4 × 24 mm^3^ subjected to the action of the concentrated forces applied on the rigid contact cylinders of the upper surface of the specimen (Figure 12).

The scaffold model consisted of 13 alternating orthogonal layers of parallel β-TCP rods. The FEM grid for the scaffold system consisted of nearly 6 million quadratic tetrahedral elements. The dimensions of the elements are not uniform throughout the model. Element size is around 100 μm for most of the model, but much smaller within the middle bottom region depicted in Figure 12, in which elements at the external surfaces of the ceramic rods have a characteristic size of ~5 μm. This refined mesh is located within the region under highest tensile stresses where fracture initiates.

An isotropic elastic behavior was assumed for the entire system since there was no experimental evidence of any plasticity prior to the fracture of the ceramic skeleton in the load-displacement curves of the composite scaffolds. The elastic moduli obtained from the instrumented indentation tests were used as input parameters for the simulation of each individual material (Table 1). The simulation of bare structures was made by selecting a negligible elastic modulus for the infiltrating material. The algorithm assumes an infinite scaffold-infiltrate interfacial strength. The following boundary conditions were selected: the two bottom contact cylinders were fixed while the top ones are free to move under a concentrated load in the direction perpendicular to the rod axes.

## 4. Conclusions

This work’s results provide further confirmation that dense hybrid ceramic/polymer composite biomaterials obtained by polymer-impregnation of bioceramic preforms are promising candidates for bone substitution and regeneration as they can exhibit mechanical performance similar to human cortical bone, both in terms of strength and toughness. The strengthening and toughening effects of polymer infiltration reported previously under compressive stresses [16–18] are not only reproduced in bending but become even more accused under this most deleterious type of solicitation. The improved enhancement in the strength is attributed to an increase on the effectiveness of the stress shielding mechanism under this configuration. The presence of the polymer in the macropores, even a relatively low-modulus polymer as PCL, increases significantly the opposition of the structure against flexure, which greatly reduces the tensile stresses in the bioceramic rods (by half in the case of PCL, and by greater factors for stiffer polymers).

Defect healing mechanism remains active and is deemed unaffected by the change in the type of solicitation. The fracture mode under the loading configuration studied here (Figure 12) is also similar to that observed in compression with crack initiation and propagation on the longitudinal rods close to the joints with the transversal ones, where the maximum tensile stresses are located.

Bending tests provide a much better means than compression to analyze the toughening mechanisms involved in improving the fracture energy (or strain energy density) on impregnated scaffolds. A significant part of the huge fracture energy increase produced upon polymer infiltration stems from the associated enhancement in strength. However, there is another important mechanism associated to the dissipation of energy by elongation of polymeric macro- and micro-fibrils that bridge the crack opening. Microfibrils generated from the polymer within the rod’s micropores produce a continuous crack microbridging toughening, while macrofibrils generated from the polymer in the macropore channels can bridge the crack wake even after very large strains. Unfortunately macrofibrils are only observed in ductile polymers (like PCL), but there is evidence of microfibrils (if shorter) even in a relatively brittle polymer as PLA. Optimal infiltrating material would obviously be one biodegradable polymer combining the stiffness of PLA, to enhance stress shielding strengthening, and PCL ductility, to provide improved toughening.

## Figures and Tables

**Table 1.** Elastic modulus and Vickers hardness determined from instrumented indentation for the individual polymers and the robocast structure rods, before and after polymer infiltration. Average values are shown with standard deviations as errors.

Material	*E* (GPa)	*H* (GPa)
PCL	0.53 ± 0.03	0.055 ± 0.004
PLA	4.52 ± 0.03	0.24 ± 0.01
TCP (Rod)	16 ± 2	0.7 ± 0.1
TCP/PCL (Rod)	19 ± 2	0.8 ± 0.2
TCP/PLA (Rod)	27 ± 3	1.7 ± 0.2
